# How Glycerol and Water Contents Affect the Structural and Functional Properties of Starch-Based Edible Films

**DOI:** 10.3390/polym10040412

**Published:** 2018-04-08

**Authors:** Ewelina Basiak, Andrzej Lenart, Frédéric Debeaufort

**Affiliations:** 1 Department of Food Engineering and Process Management, Faculty of Food Sciences, Warsaw University of Life Sciences-SGGW (WULS-SGGW), 159c Nowoursynowska St., 02-776 Warsaw, Poland; andrzej_lenart@sggw.pl; 2 Food and Wine Physical Chemistry Lab, University Bourgogne Franche-Comté, UMR A02.102, 1 Esplanade Erasme, 21000 Dijon, France; frederic.debeaufort@u-bourgogne.fr

**Keywords:** starch films, glycerol, hydration properties, surface and functional properties, molecular interactions

## Abstract

As starch is an inexpensive, filmogenic, easily processable and a widely available material, it is a material that can be utilized in the creation of biodegradable films and containers, presenting as a viable alternative to polymers derived from petrol. Moreover, starch could also be used to create edible coatings for fresh foods in order to extend shelf life. As such, wheat starch films with two glycerol contents were formulated to mimic the effects of compounds currently used to coat fruit. Their structural and functional properties were characterized. This study found that the transfer properties of starch films containing 33% of plasticizer was less effective than film comprised of 50% glycerol. Water diffusivity, oxygen permeability, and water vapor permeability at two different humidity gradients, surface tension, works of surface adhesion and cohesion, and moisture sorption were tested. Glycerol content does not play a significant role on the color or mechanical properties. This work shows that glycerol can strongly affect the functional properties of starch-based coatings and films.

## 1. Introduction

One of the major drawbacks to the widespread use of packaging produced from petrol is its environmental impact. An alternative to plastic, therefore, should be sought, and the development of biodegradable films and coatings made from natural bio-sourced polymers should be encouraged. In the 1980s, starch was discovered to be a filmogenic material, and while some basic research on the potential of this was conducted, scientists chose to focus more instead on other (more expensive and limited) biopolymers such as proteins. Nowadays, due to its accessibility and low production costs, films made almost entirely from starch are now emerging as an excellent solution to the problem of contemporary society’s overreliance on plastic packaging, and can potentially be used for a variety of food-stuffs, including fresh fruit, in addition to cosmetic or pharmaceutical products. Such packaging materials can be manufactured for major investment [1,2], influencing a reduction in the amount of crude oil-based packaging produced and used. Moreover, in contrast to synthetic polymers, starch-based films and coatings are edible and could enable a revolution in global packaging/food markets. Due to the biodegradable nature of starch films, they breakdown significantly faster than foils produced from crude oil. Not requiring special composting conditions, a starch film could potentially breakdown in less than a few weeks. Similarly, food coated in a starch-based film would only require a light washing in tap water in order to be ready for consumption due to its hydrophilic character. As such, given the potential of this material to revolutionize the packaging and preservation of food, it is important that we fully understand the limitations of starch-based films and coatings in addition to their numerous advantages.

The annual supply of starch is estimated to be approximately a billion tons in developed and developing countries. The most commonly produced starch is that from maize, followed by wheat starch, and potato and rice starches. Despite the dominance of maize starch globally, wheat starch is the most prevalent starch-type produced in Europe due to its availability, versatility, and low production cost [3,4]. Used as a thickener, texturizer, gelling agent, adhesive and moisture-retainer, starch’s processibility and film-forming ability means that it can be combined with water to form a continuous polymeric entangled phase, or a plasticizer to increase flexibility by a reduction in intramolecular hydrogen bonding along polymer chains, thereby increasing the intermolecular spacing [4,5,6,7]. The plasticizing system can also be constituted of two or more components [8]. When several plasticizers (other than water) are presented in the system, strong plasticizer-plasticizer or plasticizer-water interactions are observable. These interactions can be cooperative in character and improve the functional properties of biodegradable materials [7]. The tensile strength of starch films without plasticizers are high, but the films are brittle and exhibit little or any elongation [9]. Thus, the use of a plasticizer is always required to produce a usable film. While water is the most effective and commonly used and plasticizer for starch, many other substances are used to plasticized this biopolymer. Generally, hydrophilic, low molecular weight polyols (glycerol, sorbitol, xylitol, polyethylene glycols, etc.) are used, in addition to compounds containing nitrogen (urea, ammonium derived chemicals, amines) and non-volatile acids such as citric or lactic or tartaric [7,10]. Polyols tend to adsorb water that depends on their molecular weight and number of hydroxyl groups [11]. The most commonly used is glycerol as it is considered non-toxic and is, therefore, well suited for use in the food industry [12]. It is compatible with amylose and could interfere with amylose chain packing [13]. Glycerol and other water-compatible diluents have plasticizing rather than antiplasticizing effects on the mechanical properties of glassy composite systems; biopolymer-based edible films [13,14], and those with a glycerol content ranging from 10% to 20% were notflexible enough for glycerol to produce varying effects depending on its concentration. Heydari et al. [15] studied the wettability properties of corn starch films. They discovered that films comprising of less than 10% glycerol were brittle and those with a glycerol content ranging from 10% to 20% were not flexible enough for effective use. The concentration of glycerol depends on the targeted applications; for instance the minimum amount of glycerol for acceptable mechanical resistance and heat sealability is about 30% for sago starch films. Abdorreza et al. [16] reported that glycerol levels below 30% could decrease the ductility of sago starch films. When the amount of glycerol exceeded 50%, the plasticity increased largely as well as its permeability. The higher the glycerol content, the higher the ductility [17], but the lower the barrier and solubility efficacy.

Therefore, based on available knowledge and new techniques (comparing to previous experiments of other authors conducted several years ago) the objective of this work was to study the influence of plasticizer content on the physical, chemical, and functional properties of films made from wheat starch and designed to be used to coast fruit, related to hydration behavior of the system.

## 2. Materials and Methods

### 2.1. Materials

Wheat starch (25% of amylose) supplied by Hortimex (Konin, Poland), anhydrous glycerol (99.9% pure) from Sigma-Aldrich (Darmstad, Germany) and 10saturated salt solutions (all Prolabo, Fontenay-sous-Bois, France) were used for fixing a wide range of relative humidities (RH) at 25°C: calcium chloride (~3% RH), lithium chloride (11%), potassium acetate (22%), magnesium chloride (33%), potassium carbonate (43%), magnesium nitrate (53%), sodium nitrite (65%), sodium chloride (75%), ammonium sulphate (81%), and ammonium dihydrogenophosphate (93%).

### 2.2. Preparation of Starch Films

Wheat starch film-forming solutions were prepared by dissolving 5 g of wheat starch powder into 100 mL of distilled water. The solutions were heated in a water bath at 85 °C for 30 min at 700 rpm, stirring to obtain solubilization and gelatinization of the starch. Then, these film-forming solutions were cooled down to 40 °C. The plasticizer was added at a weight ratio of 0.5:1 and 0.3:1 glycerol: starch while stirring at 150 rpm. The concentration of plasticizer was chosen according to the results of preliminary experiment, i.e., a minimum concentration of 30% is required so that the film is ductile and does not become brittle, and a maximum of 50% as with higher glycerol contents, the films become soggy and white (overplasticized). A defined volume of film-forming solution was poured into a Petri dish to obtain a constant film thickness regardless of the composition. Films were dried at 25 °C and 30% relative humidity (RH) for 48 h in a climatic chamber (KBF 240, Binder, Tuttlingen, Germany). Dry films were peeled off and stored at 53 ± 1% RH and 25 ± 1 °C in desiccators containing saturated magnesium chloride for at least 7 days prior to any testing.

Films without glycerol were very brittle and could not be analyzed as they all broke during testing.

### 2.3. Film Characterizations

#### 2.3.1. Film Thickness Measurements

Film thickness was measured with a PosiTector 6000 (DeFelsko, Ogdensburg, NY, USA) digital micrometer to the nearest 1 µm overa 0–100 µm range. Prior to film thickness measurements, the electronic gauge was calibrated at 74 and 139 µm using standards. The thickness of each film was measured in five places, once in the center of the film and in four places along its perimeter, and an average value was used in the calculations.

#### 2.3.2. Film Observations: Colour and Environmental Scanning Electron Microscopy (ESEM)

The color of the films was determined using a colorimeter (Minolta, Model CR-300, Osaka, Japan) using the CIE LAB color parameters: *L*, from black (0) to white (100); *a*, from green (−) to red (+); and *b*, from blue (−) to yellow (+) [18]. The color of the films was expressed as the total color difference (∆*E*) according to the following equation [19]:

(1) $$\Delta E=\sqrt{{\left(L-L*\right)}^{2}+{\left(a-a*\right)}^{2}+{\left(b-b*\right)}^{2}}$$

where: L*, a* and b* are the color parameter of a white standard (L* = 96.74, a* = 0.09, b* = 2.20) used as the film background.

Film microstructure was observed using an environmental scanning electron microscope (ESEM, Philips XL 30 ESEM, Tokyo, Japan). A 0.5 × 1.0 cm^2^ film was fixed on the support using double sided adhesive at an angle of 90° to the surface, which allowed the observation of the cross-section of the film. All film samples were cut with a new razor blade in an attempt to minimize morphological damage. Films were focused up to ×15,000, and magnifications ranging from ×800 to ×8000 where selected, with an intensity of 8kV and absolute pressure of 230 Pa in presence of water (RH~30% at 5 °C). No special preparation, such as palladium or gold coating, was necessary for ESEM observations.

#### 2.3.3. Fourier Transform Infrared with Attenuated Total Reflection (FTIR-ATR)

The Fourier Transform Infrared spectra from each film were obtained using a spectrometer (IFS 28, Brucker, Coventry, UK) using Attenuated Total Reflectance (ATR) using ZnSe crystal. All the spectra were an average of 64 scans at a resolution of 4 cm^−1^, from 650 to 4000 cm^−1^ and determined at 25 °C on films equilibrated at 53% RH. This analysis was undertaken to determinate the modifications at the molecular scale of the surface induced by the glycerol addition.

#### 2.3.4. Thermal Properties

The thermal stability of films was determined by Thermo-Gravimetry Analysis (TGA). Films stored at 53% RH and 25 °C were scanned using a thermogravimetric analyzer (TGA-7, Perkin Elmer, Norwalk, CT, USA) from 40 to 800 °C at a rate of 10 °C/min. Nitrogen was used as the purge gas at a flow rate of 20 mL/min; nitrogen also prevented any chemical degradation such as oxidation.

#### 2.3.5. Surface Properties

The contact angle (θ) was measured at the triple contact point of liquid/solid/air phases. It describes the relationship between the surface tension energies of three phases according the Young equation [20].

The sessile drop method was used for measuring the contact angle: a droplet (about 1.5 µL) of a test liquid was placed on a horizontal film surface. Measurements were done using a DGD-DX goniometer equipped with DIGIDROP image analysis software (GBX, Romans-sur-Isere, France), following Karbowiak’s et al. [21] methodological approach. The contact angle was measured on both sides of the drop and averaged. The contact angle and drop volume kinetics were carried out over 120 s. The analysis of kinetics determined the metastable contact angle (pseudo-equilibrium) and measured the rate of absorption as well as swelling of the film’s surface. The effect of evaporation was assessed on aluminum foil, considered to be an impermeable reference surface, and subtracted from the sample. Then, the rate of evaporation was examined with the data collected in the study of kinetic wetting and absorption. Measurements for all samples were done on the side of the films exposed to air during drying with the aim of preventing the support (Petri dish) effect.

The estimation of the critical surface tension $(\gamma_{c}$ of the starch-based films was obtained from the Zisman plot [22] as previously described by Basiak et al. [23]. Cyclopentanol, diiodomethane, ethylene glycol, glycol, methyl benzoate, n-octane, polyethylene glycol, tetradecane, water, and 1-bromonaphtalane were selected as the liquids as the surface tension properties, dispersive and polar components of each are known. These liquids were also used for the determination of the surface tension and its components using the Owens and Wendt method [24]. Indeed, the surface tension of films $\gamma_{c}$ and their dispersive $\gamma_{S}^{D}$ and polar $\gamma_{S}^{P}$ components were calculated from the Equations (2) and (3):

(2) $$\gamma_{c}=\gamma_{S}^{P}+\gamma_{S}^{D}$$

(3) $$\gamma_{L}=\left(1+\cos\theta \right)=2\left(\sqrt{\gamma_{L}^{P}\gamma_{S}^{P}}+\sqrt{\gamma_{L}^{D}\gamma_{S}^{D}}\right)$$

In Equation (3) $\gamma_{L}^{P}$ and $\gamma_{L}^{D}$ are unknowns so it is insufficient to determine the $\gamma_{c}$ of a polymer surface. Thus, the contact angle has to be measured using at least two liquids, knowing their respective surface tensions and their dispersive $\gamma_{S}^{D}$ and polar $\gamma_{S}^{P}$ components. With these properties known, Equation (2) can be utilized.

Work of adhesion (per unit area, W_a_), work of cohesion (per unit area, W_c_), and spreading coefficient (for a liquid over a solid, W_s_) were calculated using Dupre (1869) equations:

(4) $$W_{a}=W_{a}^{P}+W_{a}^{D}↔2\left(\sqrt{\gamma_{L}^{P}\gamma_{S}^{P}}+\sqrt{\gamma_{L}^{D}\gamma_{S}^{D}}\right)=\gamma_{L}\left(1+\cos\theta \right)$$

(5) $$W_{c}=2\gamma_{LV}$$

(6) $$W_{s}=W_{a}-W_{c}=\gamma_{SV}-\gamma_{LV}-\gamma_{LS}$$

#### 2.3.6. Hydration Properties

The water content was measured by determination of the weight loss of the film after drying at 105 °C for 24 h on films after equilibration above the saturated salt solutions. All samples were performed in triplicate. The amount of water absorbed was expressed as grams of water per grams of dry matter.

The sorption isotherm of films was determined as 25 °C. Samples of films were cut into small pieces (2 × 2 cm²) and weighed to the nearest 0.0001 g in pre-weighed vials. Films were stored up to equilibrium in desiccators with fixed relative humidity (from ~3% to 93%) in triplicate, and equilibrium checked over 9 months. The 10saturated salt solutions were used for fixing the relative humidities. The final (equilibrium) water content was checked by drying the films at 105 °C for 24 h. From the kinetics of water sorption, the diffusivity of water in films was calculated according the Crank’s solution of the 2nd Fick law.

Sorption isotherms of water vapor were fitted with the Guggenheim-Anderson-de Boer (GAB) model (Equation (7)), for water activities up to 0.85:

(7) $$m=\frac{m_{0}\cdot C\cdot K\cdot a_{w}}{\left(1-K\cdot a_{w}\right)\cdot \left(1-K\cdot a_{w}+C\cdot K\cdot a_{w}\right)}$$

where: *m* is the water content at equilibrium, $m_{0}$ is the water of water related to the monolayer, *a_w_* is the water activity of the sample, and *C* and *K* are constants related to the sorption enthalpy of the first and of subsequent layers, respectively. The GAB model was fit to experimental values using the Table-Curve 5.1 software (Systat, Chicago, IL, USA).

Moreover, the diffusivity was obtained from the water vapor sorption kinetics (from sorption isotherm determination), assuming the following hypothesis. When the apparent diffusivity Dapp is constant and independent of the concentration, the transfer rate through a sheet of thickness 2L (m) stored in an atmosphere of infinite volume with a constant concentration of diffusing substance (Ceq, kg/m^3^) and with an infinite mass convection coefficient can be expressed by Equation (8) [25]:

(8) $$\frac{\partial C}{\partial t}=D\frac{\partial^{2}C}{\partial X^{2}}$$

The initial and boundary conditions for sorption experiment are given in Equations (9)–(11). Equation (12) is obtained by integrating Equation (9) for a sheet of 2L thickness [25]:

At *t* = 0, *C*_0_ = 0 = for 0 ≥ *x* ≥ *L*; (9)

At *t* ≥ 0, *C_t_* = *C*_max_ for *x* = *L*; (10)

And at *t* = ∞, *C*_∞_ = *C*_max_, for 0 ≥ *x* ≥ *L*

(11) $$\frac{\partial C}{\partial X}=0;\;x=0;\;t\geq 0$$

(12) $$\frac{C_{t}-C_{0}}{C_{max}-C_{0}}=\frac{M_{t}}{M_{\infty }}=1-\overset{\infty }{\underset{n=0}{\sum }}\frac{8}{{\left(2+1\right)}^{2}\pi^{2}}\exp\left[-\frac{{\left(2n+1\right)}^{2}\pi^{2}}{4L^{2}}Dt\right]$$

where *t* is the time (s), Mt the amount of water vapor sorbed by the sheet over time *t*, and *M*_∞_ is the maximum amount of water sorbed at equilibrium (theoretical infinite time). Apparent water diffusivity within the films was estimated by fitting Equation (12) to the experimental release kinetic data using the NLIN procedure of SAS after a pre-estimation of D using Excel. The equation was fit with *n* = 5.

The swelling index was measured to assess the impact of immersion on water absorption. The samples were prepared from five films produced of the same composition. They were cut into 2 × 2 cm^2^ pieces and weighed. They were then immersed in distilled water (25 °C) for 2 min. Wet samples were wiped with filter paper to remove excess liquid and weighed. The amount of adsorbed water was calculated in percentages. The measurement was repeated for each type of film three times, and the average was taken as the final result.

The water solubility was determined according the Gontard et al. [26] method. Films were cut into 2 × 2 cm^2^ pieces dried at 105 °C for 24 h and weighted. Films were individually placed in 50 mL beakers filled with 20 mL of distilled water, capped and stored at 25 ± 1 °C for 24 h. Film pieces were then taken out and dried at 105 °C for 24 h to determine the final weight of dry matter. These steps were repeated three times. Loss of total soluble matter was calculated from the initial and final dry weight of films.

#### 2.3.7. Mechanical Properties

Tensile strength and elongation at break of the films were measured using a TA-XT2i Texture Analyser (Stable Microsystems, Goldaming, UK) according to the ASTM D882-95 method [27]. 10 × 2.5 cm² specimens were cut with scissors. Self-tightening roller grips were used to perform tensile tests. The initial distance between the grips and the initial velocity were adjusted to 50 mm and 1 mm/s. Mechanical properties were calculated using the average thickness of each film sample and replicated 10 times.

#### 2.3.8. Water Vapor (WVP) and Oxygen (OP) Permeabilities

Water vapor permeability of films was measured gravimetrically according to Debeaufort et al. [14] who adapted the ASTM E96-80 [28] standard method to hydrophilic edible films and coatings. Film samples were placed between two rubber rings on the top of glass cells containing silica gel, sodium chloride or distilled water allowing obtaining internal relative humidities (RH) of the permeation cells at ~0%, 75%, and 100%. The permeation cells were then placed in a climatic ventilated chamber (KBF 240, Binder, Tuttlingen, Germany) maintained at a RH of 30% and temperature of 25 °C, and the weight was recorded daily for at least 10 days. Water vapor permeability was calculated using the following equation:

(13) $$\mathrm{WVP}=\frac{\Delta m\cdot e}{A\cdot \Delta t\cdot \Delta p}$$

where: Δ*m*/Δ*t*—weight of moisture loss per unit of time (g/s), *A*—film area exposed to moisture transfer (8.04 × 10^−4^ m^2^), *e*—film thickness (m), Δ*p*—water vapor pressure differential between the two sides of the film (Pa). Measurements were performed at least three times for each RH differential tested.

Oxygen permeability was measured using the manometric method according the ISO 15105-1 Standard using the Brügger equipment, Type GDP-C (Brügger Feinmechanik GmBH, Munich, Germany).

The test chambers of the permeation cell were first degassed under a vacuum, then the upper side was swept by a humidified oxygen flow at a rate of about 80 mL/min at atmospheric pressure. The increase in pressure in the downside chamber during the test period was assessed and displayed by an external computer. Data were recorded and permeance was calculated by GDP-C software (with temperature compensation). The sample temperature (25 °C) was adjusted using an external thermostat (HAAKE F3 with Waterbath K). The desired RH was regulated in an external saturation system (53% and 75% RH), so humidified oxygen gas circulated in the permeation cell.

### 2.4. Statistical Analysis

Statistical analysis was performed with Statgraphics Plus, version 5.0 (Manugistics Corp., Rockville, MD, USA). The analysis of variance (ANOVA) and Fisher’s LSD multiple comparisons were performed to detect significant differences in properties of films. The significance level used was 0.005.

## 3. Results and Discussion

### 3.1. Hydration Properties

**Figure 1:**
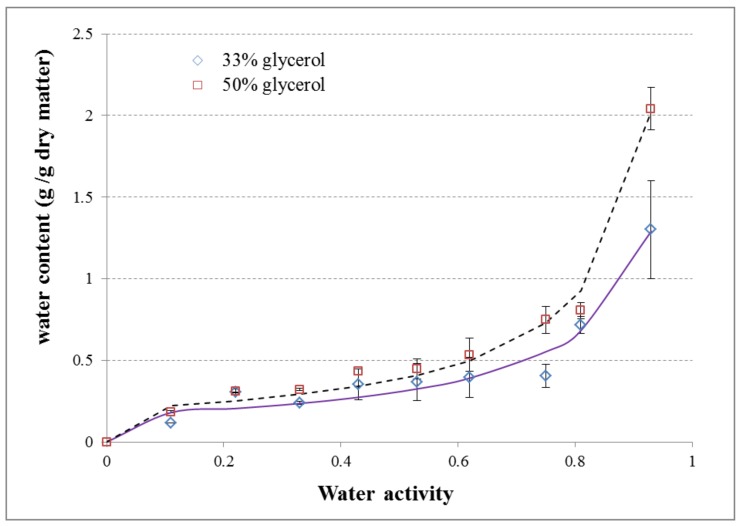
Moisture sorption isotherms of starch containing either 33% or 50% (*w*/*w*) glycerol in 5% starch solutions (g_water_/g·dm) at 25°C. Symbols are experimental values and lines values fitted by GAB model.

The moisture sorption characteristic of the films is important for predicting the stability of the films during storage, since the shelf life of the biodegradable packages in different storage conditions is dependent on their moisture uptake. The water sorption isotherms of films prepared with different plasticizer concentrations, i.e., 33% and 50% of glycerol, are shown in Figure 1, while the GAB parameters (m_o_, C, K) and correlation coefficients (R^2^) are given in Table 1.

Below *a_w_* = 0.55, the water was strongly involved in the structural organization of a biopolymer network. Bound water is located in the immediate contact of the starch chains, has a reduced activity. Interactions occurring between starch and glycerol may promote adsorption of moisture as displayed in Figure 1, due to changes of dimension in the area where the adhesion between interfaces (glycerol-starch) is weak, thus creating a step that simplifies the accumulation of water molecules [29]. As the glycerol content increases the moisture absorption increases too.

In the range of *a_w_* = 0.60–0.75 the highest difference of water content between films with 33% and 50% of glycerol was visible. Figure 1 shows the effect of glycerol incorporation to the starch matrix. Simultaneously, in experimental data, the higher relative humidity was the strongest influence of plasticization. According to higher water activity, glycerol increases the water content in starch film matrixes; however, under these conditions, hydrophilic polymeric chains swell, altering its structure.

The GAB model was represented well the experimental data, as previously reported by other authors [30,31,32,33]. Films with a higher plasticizer content have a higher monolayer water content (Mo). These results may be due to glycerol’s high hygroscopicity. Glycerol molecule presents upper water affinity, demonstrated by sorption isotherms presented in the literature [34]. Kibar and Us [35] reported that in starch films, plasticizers are generally more hygroscopic than starch. Indeed, Muscat et al. [36] and Talja et al. [37] showed that the amount of water sorbed by starch films increases with the polyol content. Thus, the difference in the water adsorption capacity of starch films (when the amount of starch is constant) is mostly dependent on the concentration of the plasticizers added. Müller et al. [38] also observed this of cassava starch films. The value for the C parameters, relative to the subsequent layer of moisture, decreased with the concentration of polyol. Moreover, the GAB model provided a good fit to the experimental data for all samples, with R^2^ values >0.98 [39]. However, both samples (with 33% and 50% of glycerol) exhibited similar behavior. The values presented in this study for the GAB constants were in agreement with the values reported in the literature [38,40,41].

Another important property of films for food packaging applications is their solubility in water. Some latent uses may require water insolubility to enhance product integrity and water resistance [42]. The difference in water solubility between the films was not significant (Table 1), however, a slight increase of solubility is observed for the 50% glycerol content film. This is not proportional to the glycerol content which means the interaction between starch and glycerol rises with the glycerol content. Farahnaky and Saberi [42] have indicated that the addition of glycerol increased the water solubility of wheat starch films from 11.7% to 27.7% when glycerol content roses from 30% to 50% (*w*/*w*). In that case, it was supposed that hydrogen bonds stabilizing the starch network were disrupted, reducing the cohesiveness of the starch matrix and then increasing its solubility in water. Ghanbarzadeh et al. [43], Hu et al. [44] and Laohakunjit and Noomhorm [45] reached similar results when testing corn, rice, and potato starch films.

The swelling index and water content (Table 1) were not significantly different between films with 33% and 50% of glycerol. This was mainly due to the amount of plasticizer we used to complete the plasticization of the starch network. Full plasticization is often related to the swollen matrix. Thus immersion in water did not additionally increase plasticization or swelling of the tested films. In previous works Basiak et al. [23,46,47] demonstrated that only the addition of protein isolate or rapeseed oil had a significant influence on the swelling index and water content of wheat starch films.

### 3.2. Interactions Involved in Starch-Glycerol Films Displayed by FTIR

**Figure 2:**
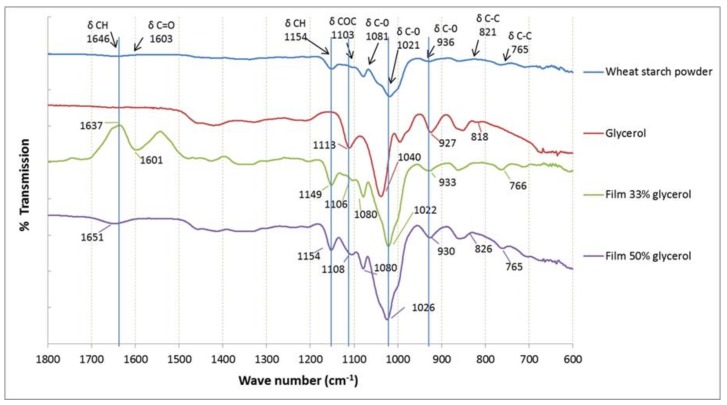
Fourier Transform Infrared (FTIR) spectra for wheat starch powder, pure glycerol, starch films containing 33% of glycerol, starch films containing 50% of glycerol. The blue vertical lines correspond to shifted peaks.

The most important intermolecular interaction determining the properties of the starch films is the hydrogen bond. Changes in the hydrogen bond network due to changes in the matrix composition alter this structure and, consequently, modulate the matrix network [4]. The FTIR spectra of glycerol-starch films (33% and 50% plasticizer content) and that of pure glycerol are presented in Figure 2. The broadband located at 3430 cm^−1^ (not showed in the graph) was conclusive and corresponded with vibrations models of intramolecular OH-groups from the absorbed water, from glycerol and from the starch polymer as also displayed by Jiménez et al. [48]. The peak located at 2928 cm^−1^ was weak and related with the vibration of the hydrogen bond. The absorption bands at 1655 and 1373 cm^−1^ were also weak and related to the CH and C=O groups. The broad bands located at 1165 and 1084 cm^−1^ had a strong character and were assigned to δCH and δCOC. The peak with wavenumber 980 cm^−1^ was stark and correlated with vibrations of δC–O [49]. Bands located at 1165–980 cm^−1^ were the typical region of saccharides. These bands turned out to be the most intensive absorbances in the IR-spectra [50].

The FTIR spectrum of pure glycerol shows five typical absorption bands located at 800 up to 1150 cm^−1^, corresponding to the vibrations of C–C and C–O linkages. Three broad bands at 850, 925, and 995 cm^−1^ correspond to the vibration of the skeleton C–C; the peak at 1045 cm^−1^ is associated to the stretching of the C–O linkage in C1 and C3, and the bond at 1117 cm^−1^ is corresponded to the stretching of C–O in C2 [51]. The effect of glycerol and water interactions can be analyzed by comparing the spectra of wheat starch powder to the spectra of starch films with 33% and 50% of glycerol. As can be seen from Figure 2, compared to the IR spectra of reference samples (wheat starch powder), characteristic peaks for saccharides were shifted. Peaks at 1014 cm^−1^ could be assigned to C–O stretching and they were shifted to 1020 cm^−1^ (starch films with 33% of glycerol) and 1023 cm^−1^ (films with 50% of glycerol). Furthermore, the characteristic peak at 2908 cm^−1^ of pure glycerol (due to C–H vibrations) was shifted to 2926 cm^−1^ for the films comprised of 33% glycerol and to 2932 cm^−1^ for the films comprised of 50% glycerol, and the peak at 3320 cm^−1^ (affiliated to O–H bond) was shifted to 3338 cm^−1^ for both films. These shifts indicated that the addition of glycerol promoted the hydrogen bonding interactions among starch and glycerol. These results proved the plasticization effect of glycerol, a finding expected due to the hydrophilic nature of glycerol and starch [52].

### 3.3. Effect of Plasticizer on Film Appearance, Colour, Microstructure and Thermal Stability

The appearance of starch films that did not contain glycerol was completely different from the other films produced. Indeed, the glycerol-free films were transparent, brittle, rough, inflexible, unpeelable, and unevenly textured. The films containing 33% or 50% of glycerol were less transparent (opalescent), though flexible and softer. Glycerol interacts with starch molecules, causing a weakening of cohesive tension. Consequently, the peeled films became more flexible. Moreover, no blooming or blushing was observed, something that occurs when the plasticizer concentration exceeds its capability limit in the polymer (overplasticization), causing phase segregation and physical exclusion of the plasticizer [45,53].

The color parameters L, a, b and total color difference ΔE of starch-glycerol films are presented in Table 1. As demonstrating in this table, an increase of the L parameter biopolymers produced more transparent films. Values obtained for wheat starch films with a glycerol content of 33% and 50% had 95.87 and 95.48 values, respectively. Glycerol slightly decreased in the value of lightness parameter. Values of red-greenish and yellow-bluish increased with plasticization. This means that films with a higher polyol content were more colored. Colour changes due to the incorporation of glycerol can be more fully demonstrated using other color functions, such as the increase in the total color difference. These results have previously been observed by Farahnaky, Saberi and Majzoobi [42] and Muscat et al. [36].

**Figure 3:**
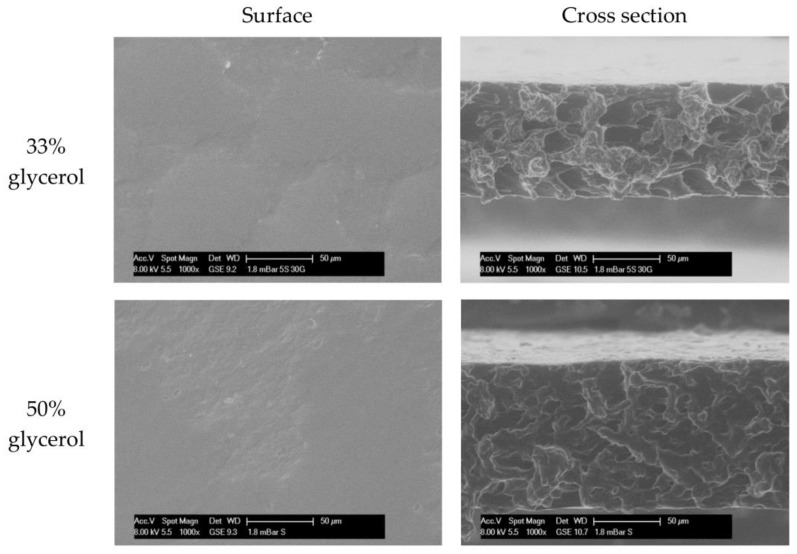
ESEM micrographs of surface exposed to air during drying and cross section of starch-based films containing 33% or 50% glycerol (*w*/*w*) at a magnification of ×1000.

Surface and cross-section images were investigated by environmental scanning electron microscopy (Figure 3). The photograph presented show the microstructure of the wheat starch-glycerol films produced. Micrographs for both films with a glycerol content of 33% and 50% showed smooth surfaces with a compact structure without pores or cracks. The similar matrix obtained by Mali et al. [54], who added 1.3% and 2% of glycerol (*w*/*w* dm) to yam starch films. The addition of polyol to starch matrix changes the microstructural arrangement of starch chains and are less dense [55]. Farahnaky, Saberi and Majzoobi [42] investigated the effect of glycerol (0, 20% and 50% of dry basis) in wheat starch films. The unplasticized films were rigid and brittle, and had cracks and pores that could potentially facilitate the transmission of the water vapor. The surface and cross-section morphologies of the plasticized films were homogeneous, but crumpled at the same time. The more plasticizer in the matrix, the smoother the films were [55]. Liu et al. [52] observed the same tendency for maize starch films with a plasticizer concentration ranging from 50% to 70% (*w*/*w*). However, the surfaces of films containing 70% polyol (*w*/*w*) were less porous with more concise networks. According to Xie et al. [10], who also measured the influence of glycerol concentration in maize films, a higher concentration of glycerol could result in a more apparent granular morphology. The homogeneous structure of starch films was a good indicator of their structural integrity, and consequently good mechanical and physical properties are expected [42].

**Figure 4:**
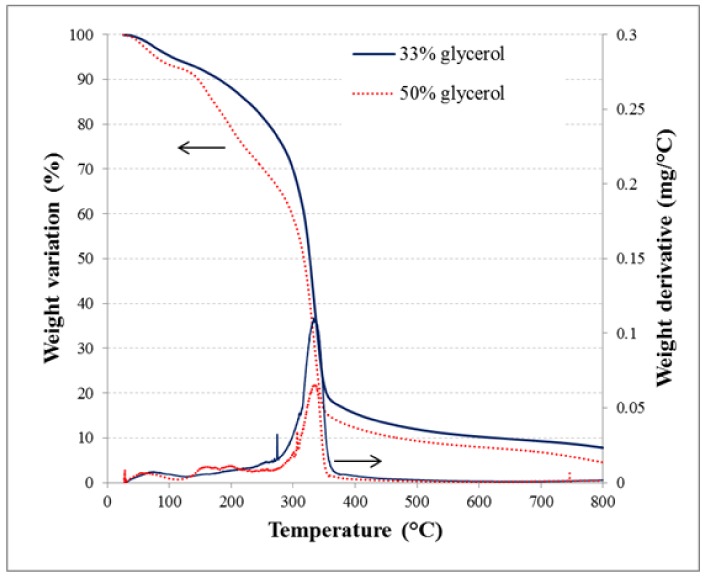
Weight loss vs. temperature (and its derivative) of films containing either 33% or 50% of glycerol equilibrated at 53% relative humidity (RH) prior thermogravimetry analysis.

An examination of how plasticizer affects the thermal behavior and viscoelastic properties of thermoplastic starch was conducted using thermal gravimetric analysis (TGA) to determine mass loss in starch matrices with a glycerol content of 33% and 50%. Figure 4 shows the weight change and its derivative according temperature. Three stages of weight loss occurred in the temperature range of 30 to 450 °C. The first stage (in the range of 30–120 °C) is attributed to water evaporation from the external surface as well as the dehydration of the intern layer. During this stage, films lost 7.7% of their weight. It could have also been related to the film water content as observed in the sorption isotherms for a 53% RH. The weight loss in the range of 120–260 °C is related to the evaporation of glycerol [56]. The incorporation of glycerol increases weight loss, as the films with containing 50% plasticizer displayed a loss about 150% higher than films containing only 33% polyols. The third zone (from 260 to 450 °C) represents the degradation of starch. The higher the glycerol content, the lower the film’s thermal stability. The TGA results clearly indicated that thermal degradation of the starch films’ relevant features converts this biopolymer into materials with useful and desirable properties.

### 3.4. Wettability Properties

**Figure 5:**
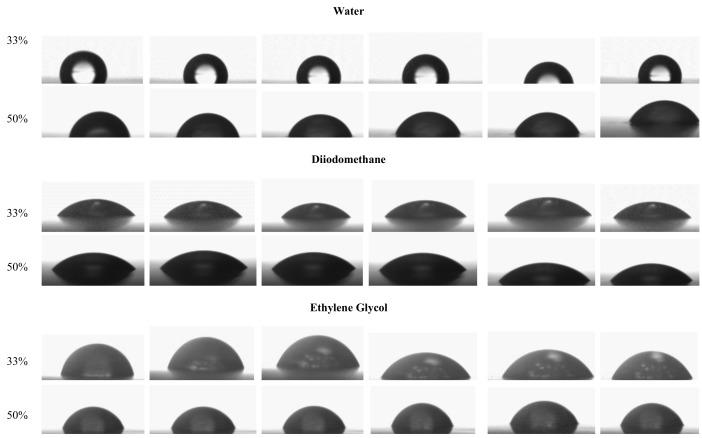

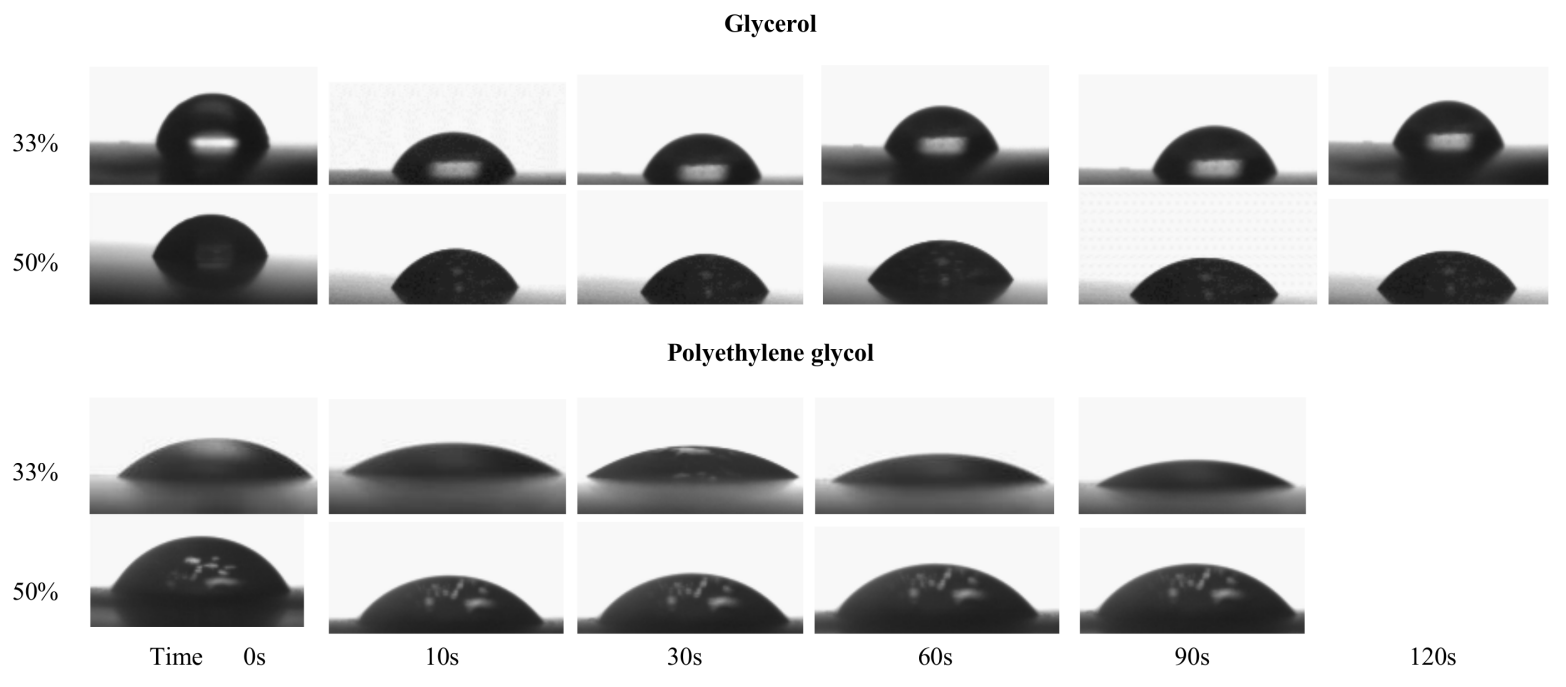
Behavior of water, diiodomethane, ethylene glycol, glycerol, and polyethylene glycol droplets on film surfaces as a function of time for starch films with 33% and 50% of glycerol.

This experiment was designed to obtain information about surface characteristics. Surface tension ($\gamma_{S}$γ_ and its components (dispersive $\gamma_{S}^{D}\gamma$_ and polar $\gamma_{S}^{P}\gamma$_), the contact angles (θ) of various liquids, and works of adhesion (WA), cohesion (WC), and spreading (WS) are given in Table 1. The contact angle is a quantitative measure of the wetting of the solid by a liquid. The value of the contact angles corresponds to the value gained at zero time, i.e., it is the initial contact angle at first droplet contact. To assess the surface properties, the contact angle kinetics of 10 liquids deposited on a film containing 33% and 50% glycerol were determined. Figure 5 shows the droplet contact angle shapes of five liquids (water, diiodomethane, ethylene glycol, glycerol, polyethylene glycol) at time: 0, 10, 30, 60, 90, and 120 s. For five other liquids: cyclopenthanol, methyl benzoate, *n*-octane, tetradecane, 1-bromonaphtalane, the spreading was instantaneous (less than 0.5 s) and the contact angle value calculated as 0°. The shape of the liquid droplets changed with the plasticizer content. In particular, water contact angles of films vary from 103° to 43° respectively for films containing 33% and 50% glycerol (*w*/*w*). The addition of glycerol reduces the contact angle and accelerates spreading. In films containing 33% glycerol, incomplete wetting of the surface occurs (contact angle is higher than 90°).

However, it is noticeable that for diiodomethane and polyethylene glycol, the contact angle is higher for films containing more plasticizer. When the shape of the drop is more rounded, this indicates that the films have better barrier properties, as absorption is not favored. Heydari et al. [15], studying the functional properties of corn starch films, observed the influence of the content of glycerol on contact angles. For films containing 25%, 30% and 35% glycerol, the water contact angles were respectively 49°, 44°, and 35°. The higher the glycerol content, the lower the contact angle.

The surface tension and its dispersive and polar components are given in Table 1. The surface tension of solid surfaces is the sum of polar and dispersive (apolar) components, indicating the nature of intermolecular interactions at the interface. The polar component is lower for the films with a 33% glycerol content than for films with a 50% plasticizer content. The higher the glycerol content, the greater the polarity of the surface, favoring hydrophilicity, water spreading, and absorption. The increased adhesion and cohesion, and decreased spreading (less energy for spreading) confirmed this.

Wheat starch films are hydrophilic as displayed from hydration properties; their surface is absorbent for moisture. It is supported by the demonstrated surface wettability and polarity. McHugh et al. [57] stated that the equilibrium of water vapor partial pressure at the inner film surface increases with the content of polyols. Water content absorption is affected by glycerol concentration; the morphology of films, as well as the swelling, significantly affects the surface properties [45].

### 3.5. Influence of Both Glycerol and Water on Functional Properties: Mechanical and Barrier Efficiencies

The Young’s Modulus (YM) is the staple test of sample stiffness: the higher the stiffness of the material, the higher the modulus of the elasticity [53]. The effect of glycerol on the modulus of elasticity as well as on tensile strength was not significant (Table 1). However, the Young’s modulus and tensile strength at break decreased with increased plasticizer content, whereas elongation increased. Talja et al. [37] observed that the addition of glycerol+sorbitol or glycerol+xylitol reduced the Young’s modulus by 14 times, resulting in a rise of 110% in elongation. Mali et al. [3,54] reported similar results for corn starch, cassava starch, and yam starch films containing two different concentrations of glycerol. Conversely, Suppakul et al. [53] did not find a significant change in elongation at break, observing instead 5.20% and 5.29% respectively for cassava starch films containing 33% and 50% sorbitol. Muscat et al. [36] observed a stronger influence of glycerol on elongation than that of xylitol on corn starch-based films. In accordance with conventional standards, packaging films should have a tensile strength of more than 3.5 MPa. It is therefore noteworthy that though there was no significant difference in the mechanical properties, only films containing 33% of glycerol satisfied the requirements and could be used as biodegradable packaging materials [15,58].

When plasticizer was incorporated into a starch matrix, it reduced the intra-molecular affinity between the starch chains by forming hydrogen bonds between plasticizer and starch molecules; thus under tensile stress, the film matrix became less dense, facilitating movements of starch chains which resulted in greater flexibility and pliability [15,59]. The range of glycerol content tested did not allow a significant modification of the film’s mechanical properties. As such, films containing 33% glycerol were flexible enough to be manipulated without risk of breaking or forming cracks. This also means that the structural properties of a film with a higher concentration of glycerol (above 33%) is not significantly different, though it is less prone to absorbing moisture. This may affect the molecular dynamics, reducing the local viscosity and increasing the diffusivity of gases and of small solutes.

Nowadays, the main goal of food packaging is the prolonging of the food’s shelf-life by minimizing any transfers between the food and its surrounding atmosphere [60]. This is why permeability determination is a key functional property for packaging materials. The water vapor permeability of all starch-based films increased with the RH gradient, as well as with the glycerol content (Table 1). Similar findings regarding the relative humidity gradient were reported by Chang et al. [61] regarding tapioca starch films. Glycerol has the biggest effect on the water content of films at the stationary state of the permeation process, which induced greater plasticization and thus favors the moisture transfer [62,63]. Farahnaky et al. [42] reported that the WVP of wheat starch films without a plasticizer was higher than that of films containing 20% or 30% glycerol. This was due to microcracks in the unplasticized films. In films containing 50% glycerol, both gradients 75–30% and 100–30% RH exhibit the same WVP values. Indeed, the glycerol content (from 33% to 50%) induced the absorption of a greater amount of water which functioned as a primary plasticizer (more efficient than the glycerol), thus increasing the permeation. These results had to be related to the diffusivity of water in films (Table 1). Indeed, from kinetics of sorption at 75% RH, the diffusivity increases doubled approximately from film containing 33% to film with 50% glycerol. Comparing our data to that of other authors, wheat starch films with both a 33% and a 50% glycerol content have a lower diffusion coefficient. Slavutsky and Bertuzzi [64] worked with corn starch with 20% glycerol content and they found that water diffusivity values were 1000 times higher than in the present work. Muscat et al. [36] obtained values of diffusivity of about 10^−13^ m^2^/s for high amylose starch films with a very low glycerol content. Müller et al. [38] also investigated the influence of glycerol on the diffusivity of cassava starch films. Films containing 0.25 *g*/*g* of dry starch have an apparent moisture diffusivity of 1.64 × 10^−11^ m^2^/s at 77% RH, whereas those containing 0.30 g of plasticizer have a 1.10 × 10^−11^ m^2^/s, and films with an additional 0.35 g of glycerol had a diffusivity value of 1.02 × 10^−11^ m^2^/s.

García et al. [1] observed a correlated dependence between glycerol content and water vapor and oxygen permeabilities. Indeed, the oxygen permeability of the films doubled when glycerol content rose from 33% to 50%, but no significant effect of the RH on oxygen transfer was observable. Oxygen permeability, therefore, mostly depends on the plasticizer ratio than on the relative humidity. Glycerol is more effective in increasing molecular mobility and in facilitating the migration of either oxygen molecules or water vapor permeability [53].

## 4. Conclusions

The influence of glycerol content and moisture levels on the physical, chemical and functional properties of wheat starch films was studied. The main conclusions drawn are as follows: films containing 33% glycerol have a lower moisture absorption capacity than films with a higher glycerol content. Films with higher plasticizer content have higher value of the monolayer water content. These results may be related with high glycerol hygroscopicity. The swelling index and water content are not significantly different between films with different glycerol content. The addition of glycerol promoted the hydrogen bonding interactions among starch and glycerol. These results prove the plasticization effect of glycerol and they are expected, considering the hydrophilic nature of glycerol and starch microstructure of wheat starch-glycerol films. When the concentration of plasticizers increased, the tensile strength and the modulus of elasticity decreased, whereas the elongation at break increased. The lower glycerol content, the better the mass transfer barrier (oxygen permeability, water diffusivity, water vapor permeability) that was obtained. Interactions between starch, glycerol, and water occur between C–O and OH groups, indicating the presence of mainly hydrogen bonds. Glycerol content does not influence significantly the color of starch films.

Micrographs, both for starch films with different glycerol content, presented smooth surfaces without pores nor cracks, and compact structures. The higher the glycerol content, the lower the film thermal stability. The TGA results clearly indicated that thermal degradation of the starch films are relevant features in order to convert this biopolymer into materials with useful and desirable properties which can be important for different packaging application. The higher the glycerol content, the greater the polarity of surface, which favors hydrophilicity and water spreading and absorption. The increase of adhesion and cohesion and the decrease of spreading (less energy for spreading) confirm this.

Properties of starch-based films strongly depend on water-glycerol contents. Altering the plasticizer content allows an increase or reduction of selected physical, chemical and functional parameters. Surface water activity and transfers parameters are key factors for fruit coating applications such as high respiration vegetables or fruits, and can be easily improved by a change in the amount of plasticizer. This work shows how glycerol and water can strongly affect the functional properties of starch-based coatings and films.

## Figures and Tables

**Table 1:** Water content, swelling index, film solubility in water, parameters of the GAB equation (m_0_, K, C), color parameters (L, a, b, ΔE), surface tension ($\gamma_{c}$), dispersive ($\gamma_{S}^{D}$) and polar ($\gamma_{S}^{P}$) components of the surface tension, critical surface tension ($\gamma_{C}$), works of adhesion (W_A_), of cohesion (W_C_) and of spreading (W_S_), tensile strength (TS), Yong modulus (YM), elongation at break (E), water vapor and oxygen permeabilities of wheat starch films containing 33% or 50% glycerol.

	Parameters		Wheat Starch Films	
			33% Glycerol Content	50% Glycerol Content
Thickness (µm)			64.1 ± 8.04 ^a^	80.8 ± 12.59 ^a^
Hydration characteristics	Water content (g_water_ 100 g^−1^·dm)		3.44 ± 0.50 ^a^	3.72 ± 0.50 ^a^
	Swelling index (%)		38.99 ± 2.44 ^a^	39.20 ± 1.43 ^a^
	Solubility in water (%)		30.16 ± 2.25 ^a^	34.76 ± 2.18 ^a^
	GAB parameters	m_0_ (g_water_ 100 g^−1^·dm)	16.4 ± 2.2 ^a^	19.8 ± 1.6 ^b^
		K	0.93 ± 0.02 ^a^	0.97 ± 0.01 ^a^
		C	754 ± 3 × 10^5^ ^a^	105 × 10^9^ ± 10^16 a^
		R^2^	0.962	0.989
Colour parameters		L	95.87 ± 0.51 ^a^	95.48 ± 0.39 ^a^
		a	−0.15 ± 0.05 ^a^	−0.24 ± 0.06 ^a^
		b	2.93 ± 0.19 ^a^	3.19 ± 0.29 ^a^
		∆*E*	0.97 ± 0.49 ^a^	1.46 ± 0.43 ^a^
Surface characteristics		$\gamma_{S}$ (mN/m)	56.22 ^a^	60.28 ^b^
		$\gamma_{S}^{D}$ (mN/m)	38.01 ^a^	35.40 ^a^
		$\gamma_{S}^{P}$ (mN/m)	18.21 ^a^	24.88 ^b^
		$\gamma_{C}$ (mN/m)	36.0 ^a^ (R^2^ = 0.87)	36.0 ^a^ (R^2^ = 0.88)
		W_A_ (mJ/m^2^)	118.5 ^a^	126.8 ^b^
		W_C_ (mJ/m^2^)	112.4 ^a^	120.5 ^b^
		W_S_ (mJ/m^2^)	−6.05 ^a^	−6.24 ^a^
Mechanical properties		TS (MPa)	3.29 ± 0.79 ^a^	2.10 ± 0.76 ^a^
		YM (Mpa)	0.12 ± 0.05 ^a^	0.10 ± 0.09 ^a^
		E (%)	15.21 ± 5.88 ^a^	18.08 ± 5.40 ^a^
Transfer properties	Water diffusivity (10^−11^ m^2^/s)	at 75% RH	4.3 ± 0.9 ^a^	7.0 ± 0.5 ^b^
	Water vapour permeability (10^−^^10^ g·m^−1^·s^−^^1^·Pa^−1^)	33–0% RH	0.52 ± 0.04 ^a^	0.92 ± 0.06 ^b^
		75–30% RH	6.05 ± 0.62 ^c^	8.77 ± 0.59 ^d^
		100–30% RH	5.48 ± 0.36 ^c^	8.01 ± 0.15 ^d^
	Oxygen permeability 10^14^ (cm^3^·m^−1^·s^−1^·Pa^−1^)	33% RH	3.58 ± 2.72 ^a^	7.23 ± 1.00 ^b^
		75% RH	4.30 ± 0.77 ^a^	7.41 ± 1.39 ^b^

Values having the same letter for a parameter are not significantly different at *p* level <0.05.
